# Effects of interfacial dynamics on the damping of biocomposites

**DOI:** 10.1038/s41598-022-23355-x

**Published:** 2022-11-21

**Authors:** Yufeng Tian, Wonsuk Kim, Alper Kiziltas, Deborah Mielewski, Alan Argento

**Affiliations:** 1 Department of Mechanical Engineering, University of Michigan-Dearborn, 4901 Evergreen Road, Dearborn, MI 48128 USA; 2 Ford Motor Company, Sustainability and Emerging Materials, Dearborn, MI 48128 USA

**Keywords:** Mechanical engineering, Nanoscale materials

## Abstract

A damping model is developed based on the mechanism of interfacial interaction in nanoscale particle reinforced composites. The model includes the elasticity of the materials and the effects of interfacial adhesion hysteresis. Specific results are given for the case of bio-based PA610 polyamide reinforced by nanocrystalline cellulose (CNC), based on a previous study that showed this composite possesses very high damping. The presence of hydrogen bonding at the interface between the particle and matrix and the large interfacial area due to the filler’s nano size are shown to be the main causes of the high damping enhancement. The influence of other parameters, such as interfacial distance and stiffness of the matrix materials are also discussed. The modeling work can be used as a guide in designing composites with good damping properties.

## Introduction

In previous work by the authors^1^, nanocrystalline cellulose (CNC) bio-based polyamide (PA610) composites have been found to possess significantly higher damping properties than polyamide controls. Based on mechanical measurements of the nondimensional damping ratio, dissipation was found to increase with decreasing CNC fiber size and increasing fiber mass fraction. Other works on similar bio-based composites have found good damping characteristics at room temperature attributed to the addition of natural fibers^2–4^, though the underlying mechanisms of the damping improvement were not studied.

There are a variety of sources of damping in polymer composites: the viscoelastic nature of the polymer, minor failure mechanisms such as the formation of cracks and the breakage of the fiber, the so-called interfacial friction, and interfacial adhesion hysteresis^5^. Parsing the contribution of the individual potential damping mechanisms is difficult. In dynamic mechanical analysis tests of polyamides, damping has been found to be relatively low and to increase due to the addition of microcrystalline cellulose^3,6^. In other cases where the base polymer has high native damping, the addition of cellulose filler has a minor effect^7^. Additionally, damping in composites attributed to crack formation^8^ and shear strain^9^ does not describe the mechanisms of damping in low load or strain applications.

The interaction of the surfaces at the interface of the matrix and filler is thought to play a role in damping. Though the effects of interfacial dynamics on damping in CNC/polyamide composites have not been studied, significant increases in damping (damping ratio or loss tangent) in carbon nanotube composites^10–12^ occur due to the large interfacial surface area between the filler and the matrix material. Moreover, increase of damping is also found in nano alumina reinforced epoxy composites due to high volume of the interphase existing between the filler and matrix^13^. However, the addition of nano-sisal whiskers to polylactic acid composites results in a decrease in damping^14^ suggesting that the size of the filler and the characteristics of both filler and matrix material are involved in the damping mechanisms.

An interfacial dynamics mechanism that could play a role in damping is due to interfacial friction. Damping increases have been found to occur in hemp fiber reinforced polypropylene^4^ due to internal friction at the interfaces. A study of interfacial friction between spider silk and a modified diamond surface shows that the friction force depends on the number of intermolecular hydrogen bonds, the relative velocity of the contacting surfaces, and the friction coefficient^15,16^. Hydrogen bonds occurring between cellulose and PA610 molecules could be a driver, separate from interfacial friction, of high damping measured by the present authors in well-bonded composites of these materials^1^. Large interfacial surface area occurring between nanoscale fillers and polymer carriers can enhance energy dissipation related to intermolecular interaction at the interface.

Interfacial mechanisms related to damping have been modelled in a few studies. In study^17^, a Prandtl–Tomlinson model describing atomic-scale friction is used to study energy dissipation using a spring-mass model. Intermolecular surface interaction resulting in “stick–slip” motion is studied for materials with periodic molecular structure. Such phenomena have been observed in atomic force microscopy experiments^18^. In study^19^, the interaction of a pair of massless materials in adhesive, elastic contact is modelled. Irreversible jumps in force state in this massless model during changes in position are described as potentially leading to energy dissipation. In study^20^, internal friction is shown to be mainly controlled by adhesion, suggesting that the “intermolecular jump” in adhesive contact is a factor in energy dissipation, and hence damping, under low load. Adhesion hysteresis is shown to be related to multiple factors^21,22^, including surface roughness, chemical heterogeneity and relative humidity. The deformable surface plays a role in accounting for adhesion hysteresis when the intermolecular force is conservative. In the case of CNC and PA610 composites, as studied in the present manuscript, hydrogen bonding strengthens the bond at the interface. Interfacial friction requires normal contact loading and interfacial sliding which is believed less likely to occur at low amplitude vibrations, compared to adhesion hysteresis produced by molecular bonding, such as hydrogen bonding.

In study^19^, the irreversibility of surface interactions is studied using an analytical model of a pair of interacting, massless materials, one of which is elastic. In some cases, moving one material toward or away from the other results in a spontaneous change in force state of the system, so-called “intermolecular jumps”, that would be accompanied by energy dissipation in a real system.

In the present work, an intermolecular contact model is developed to study the interaction of a polymer matrix material and an embedded nano-scale particle, such as what commonly occurs in nano-reinforced composite materials. The mass of the particle filler, as well as the elasticity of filler and matrix, are included in the model, along with hydrogen bonding and van der Waals forces acting between the interacting materials. The equations for the model are derived and the case of composites consisting of PA610 polyamide matrix reinforced by nanoscale cellulose fillers are treated numerically. The foci of the model are factors related to the energy dissipation in the composites including particle size, hydrogen bonding density, elastic moduli of the materials, and height of surface asperities.

## Methods

### Model

**Figure 1:**
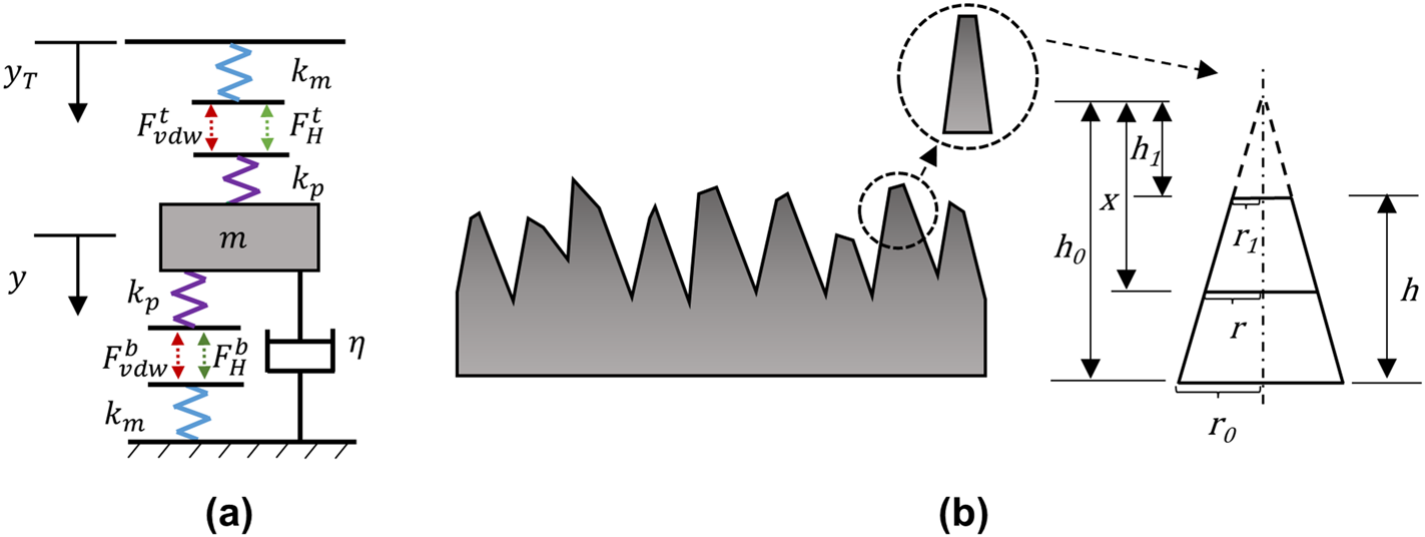
(**a**) Schematic of the model. (**b**) Schematic of the geometry of an asperity.

The specific case treated is that of a nanocellulose particle embedded in PA610 polymer, but the model is applicable to any pair of materials. The top and bottom boundaries in Fig. 1a represent the positions of the PA610 polymer surfaces, and the mass in the middle represents that of the CNC particle. Coordinate ${y}_{T}$ is the input to the system representing the motion of the bulk matrix material. The blue and purple springs represent the elastic stiffnesses of the PA610 matrix (${k}_{m}$) and the CNC particle (${k}_{p}$), respectively. The green and red dashed arrows are the hydrogen bond forces (${F}_{H}^{t}$ and ${F}_{H}^{b}$) and van der Waals forces (${F}_{vdw}^{t}$ and ${F}_{vdw}^{b}$), respectively, per unit area acting between CNC and PA610. The superscripts $t$ and $b$ denote top and bottom elements, respectively. Because ${F}_{vdw}$ and ${F}_{H}$ are independent and both exist between the two contacting surfaces, they are modeled to be parallel, and the combination of the forces becomes the total intermolecular force which will be described in detail in the section of Intermolecular force. A linear viscous damper is commonly used in the interaction between surfaces at atomic scale, such as the friction study^17^. In the current model, the damper acts as the means to characterize the amount of kinetic energy dissipated from the system. This dissipation mechanism is explained using the many-body model^23^.

The model describes the intermolecular contact in a unit area. Thus, the constants in the equation below represent quantities per unit area. The governing equation of the model is:

1 $$m\ddot{y}+\eta \dot{y}-{(F}_{vdw}^{t}+ {F}_{H}^{t})+{(F}_{vdw}^{b}+ {F}_{H}^{b})= 0,$$

where an overdot denotes time derivative, $y$ is the displacement of the CNC particle, $m$ is the mass of the particle per unit area, and $\eta$ is the damping constant per unit area which is given in terms of the mass $m$, the dimensionless damping ratio $\upzeta$, and the equivalent spring constant ${k}_{0}$:

2 $$\eta =2\upzeta \sqrt{{k}_{0}m},$$

3 $${k}_{0}=\frac{{k}_{p} \, {k}_{m}}{{k}_{p}+ {k}_{m}}.$$

In (3), ${k}_{p}$ and ${k}_{m}$ are related to the roughness of the contacting material surfaces and Young’s moduli of the particle and matrix, as described in the section of Elastic force. The elastic and intermolecular forces are related by:

4 $${F}_{vdw}^{t}+ {F}_{H}^{t}= {F}_{p}^{t}={F}_{m}^{t},$$

5 $${F}_{vdw}^{b}+ {F}_{H}^{b}= {F}_{p}^{b}={F}_{m}^{b},$$

where ${F}_{p}^{t}$ and ${F}_{p}^{b}$ are the forces per unit area in the top and bottom elastic elements of the CNC particle, respectively, and ${F}_{m}^{t}$ and ${F}_{m}^{b}$ are the forces per unit area in the elastic elements of the top and bottom matrix material, respectively. Note, in Fig. 1a and Eq. (4), the top intermolecular forces are related to ${y}_{T}$ and $y$. In the calculation of the bottom intermolecular forces, the bottom surface of the matrix is fixed, without loss of generality. Note also that all elastic elements are stretched in the initial balanced position due to the intermolecular forces.

### Intermolecular force

The intermolecular forces considered in this model are the van der Waals force and the hydrogen bonding force. Here, the van der Waals force is modeled using the Lennard–Jones adhesion potential^24^:

6 $${F}_{vdw}\left(R\right)= \frac{A}{6\pi {z}_{0}^{3}}\left[ {\left(\frac{{z}_{0}}{R}\right)}^{3}- {\left(\frac{{z}_{0}}{R}\right)}^{9}\right],$$

where $A$ is the Hamaker constant^25^ and ${z}_{0}$ is the equilibrium distance of the force. These two constants are specified for the specific materials. Note that ${F}_{vdw}(R)$ in (6) is per unit area. Here, and in subsequent equations, the intermolecular distance $R$ represents ${R}_{t}$ or ${R}_{b}$ for the top or bottom interacting surfaces, respectively. Expressions for ${R}_{t}$ and ${R}_{b}$ are given in the Supplementary Information file (Eqs. (S.1)–(S.3)).

The Dreiding force field^26^ is commonly used for hydrogen bonding, and has potential energy given by:

7 $${P}_{Hs}\left(R\right)={D}_{H}\left[5{\left(\frac{{R}_{H}}{R}\right)}^{12}-6{\left(\frac{{R}_{H}}{R}\right)}^{10}\right]{\text{cos}}^{4}\left({\theta }_{DHA}\right).$$

Here, ${\theta }_{DHA}$ which is a function of $R$, is the angle between the donor of the hydrogen atom, the hydrogen atom, and the acceptor of the hydrogen atom (see Supplementary Information file (Eq. (S.7)). ${D}_{H}$ is the minimum potential energy of the force, and ${R}_{H}$ is the equilibrium distance between the donor and acceptor atoms. The Dreiding force, ${F}_{Hs}\left(R\right)$, is:

8 $${F}_{Hs}\left(R\right)=-\frac{\partial {P}_{Hs}}{\partial R}=\frac{{60D}_{H}}{{R}_{H}}\left[{\left(\frac{{R}_{H}}{R}\right)}^{13}- {\left(\frac{{R}_{H}}{R}\right)}^{11}\right]{\cos}^{4}\left({\theta }_{DHA}\right)+{D}_{H}\left[5{\left(\frac{{R}_{H}}{R}\right)}^{12}-6{\left(\frac{{R}_{H}}{R}\right)}^{10}\right]\frac{d{[\cos}^{4}({\theta }_{DHA})]}{dR}.$$

This equation is for a single hydrogen bond. For generality, the model here is developed per unit area. The number of hydrogen bonds in a unit area is defined as ${n}_{H}$, so that (8) can be re-expressed per unit area as:

9 $${F}_{H}\left(R\right)={n}_{H}{F}_{Hs}\left(R\right).$$

It is noted that the intermolecular forces are dependent on the dependent variable $y$(t) because of the dependence of the intermolecular distances on $y$, as described in the Supplementary Information file (Eqs. (S.1)–(S.3)).

### Elastic force

When surfaces make contact, asperities deform significantly more than the bulk material. Thus, for the nanoscale of the present model, the elastic elements are modeled based on the surface asperities. Here, surface asperities are modeled as a truncated cone-shape (Fig. 1b), adapted from that in study^27^.

In Fig. 1b, ${r}_{0}$ and ${r}_{1}$ are the radii of the bottom and top circular surfaces, respectively, ${h}_{0}$ and ${h}_{1}$ are the heights of the whole cone-shaped asperity and the truncated part of the cone, respectively, and $h$ is the height of the asperity. The variable $x$ is the distance to a cross section of the asperity from the tip of the same whole cone, and $r$ is the radius of this cross section. The deformation of the asperity can be obtained by

10 $$\Delta h={\int }_{{h}_{1}}^{{h}_{0}}\varepsilon dx=\frac{f}{E}{\int }_{{h}_{1}}^{{h}_{0}}\frac{1}{{A}_{s}}dx,$$

where $\varepsilon$ is the axial normal strain of the asperity under the applied force, $f$, $E$ is Young’s modulus of the material, and ${A}_{s}$ is the area of the cross-section given by ${A}_{s}=\pi {\left({r}_{0}/{h}_{0}\right)}^{2}{x}^{2}$. Then, the relationship between the deformation Δ*h* and the elastic force per unit area, *F*_*a*_, is

11 $${F}_{a}=\frac{f}{\pi {r}_{0}^{2}}=\frac{E\Delta h}{h\frac{{h}_{0}}{{h}_{1}}}.$$

From Eq. (11) the equivalent stiffness per unit area of an asperity of height $h$ is

12 $$k=\frac{f}{\Delta h\pi {r}_{0}^{2}}=\frac{E}{L},$$

where $L=h{(h}_{0}/{h}_{1}).$ Similarly, the equivalent stiffnesses per unit area of the matrix and particle in the developed model are

13 $${k}_{p}= \frac{{E}_{p}}{{h}_{p}\frac{{h}_{\mathrm{p}0}}{{h}_{\mathrm{p}1}}}= \frac{{E}_{p}}{{L}_{p}},$$

14 $${k}_{m}= \frac{{E}_{m}}{{h}_{m}\frac{{h}_{\mathrm{m}0}}{{h}_{\mathrm{m}1}}}= \frac{{E}_{m}}{{L}_{m}}.$$

The elastic forces per unit area of the particle and matrix for the top and bottom parts are given as:

15 $$\begin{gathered} F_{p}^{t} = k_{p} \Delta h_{1} \hfill \\ F_{m}^{t} = k_{m} \Delta h_{3} , \hfill \\ \end{gathered}$$

16 $$\begin{gathered} F_{p}^{b} = k_{p} \Delta h_{2} \hfill \\ F_{m}^{b} = k_{m} \Delta h_{4} , \hfill \\ \end{gathered}$$

where $\Delta {h}_{1}$ and $\Delta {h}_{3}$ are the current deformations of the particle and matrix elastic elements above the mass, respectively, and $\Delta {h}_{2}$ and $\Delta {h}_{4}$ are the current deformations of the particle and matrix elastic elements below the mass, respectively.

### Material properties

Damping results quoted in the Results and discussion section are from Ref.^1^. They are for PA610 composites filled with: (i) cellulose nanocrystal (NVC 100; manufactured by CelluForce) which is specified to have dimensions from 2.3 to 103 nm, but form particles from 1 to 50 μm; (ii) 4 μm and (iii) 100 μm cellulose (VIVAPUR CS 4 FM, VIVAPUR 102; both manufactured by JRS Pharma). The matrix material is PA610 bio-based nylon resin (BASF).

### Parameters

The parameters used in the model, and their corresponding sources, are summarized in Table 1. The values in the model are for the case of CNC reinforced PA610. The mass per unit area, $m$, of the CNC particles is derived from the density and size of the spherical particle (Diameter, $D$ = 100 nm) from the manufacturer’s material specification literature. In the case of unit contacting surface area, the mass per unit area of the particle is approximately $m=D\rho$, where $\rho$ is the density of the particle from the manufacturer’s material specification literature. The ratio of ${h}_{p0}$/${h}_{p1}$ and ${h}_{m0}$/${h}_{m1}$ are set as 3, based on transmission electron microscopy of similar materials^28^. Note that results will be given for a range of parameter values.

**Table 1:** Parameters.

Parameters	References
$m$ = 1.5e−22 kg/nm^2^	*
$\rho$ = 1.5 g/cm^3^	*
${E}_{m}$ = 1.4 nN/nm^2^	**
${E}_{p}$ = 145 nN/nm^2^	^29^
${h}_{m}$ = 0.5 nm	^30,31^
${h}_{p}$ = 0.5 nm	^30–32^
$A$ = 8.45e−20 J	^33,34^
${Z}_{0}$ = 0.343 nm	^26,35^
${D}_{H}$ = 9.5 kcal/mol	^26^
${R}_{H}$ = 0.276 nm	^26^
${n}_{H}$ = 2 nm^−2^	^36^
$\theta$ = 165°	^37^
${L}_{ab}$ = 0.95 nm	^26^
$\zeta$ = 0.3	^17,38^

*Calculated from company specifications.

**Experimentally measured by the authors.

### Numerical solution

The governing equations for the model are (1), (4–6), (8), (9), (15), (16), (S.2) and (S.3). These form a system of 17 coupled, nonlinear differential equations (note that each of Eqs. (4)–(6), (8), (9), (15), (16) represents one equation for the top and one for the bottom segments of the model) for ${F}_{vdw}^{t}$, ${F}_{H}^{t}$, ${F}_{p}^{t}$, ${F}_{m}^{t}$, ${F}_{vdw}^{b}$, ${F}_{H}^{b}$, ${F}_{p}^{b}$, ${F}_{m}^{b}$, ${F}_{Hs}^{t}$, ${F}_{Hs}^{b}$, ${R}_{t}$, ${R}_{b}$, $\Delta {h}_{1}$, $\Delta {h}_{2}$, $\Delta {h}_{3}$, $\Delta {h}_{4}$, $y$. Solving these directly using a common numerical technique such as the Runge–Kutta method is computationally prohibitive. Here, a predictor–corrector-method, called “velocity Verlet,” widely used in molecular dynamic simulations, is applied for the calculation^39^. The solution procedure is as follows. The first step is setting $y$ = 0 and giving an initial input to ${y}_{T}$ in the initial time step. Then, $\Delta {h}_{1}$ is substituted from Eq. (S.2) into both of (15). These expressions, along with (6), (8), (9) (for the top segment), are substituted into Eq. (4), giving two coupled equations for ${R}_{t}$ and $\Delta {h}_{3}$ from which ${R}_{t}$ and $\Delta {h}_{3}$ are determined at this instant. Simultaneously, ${R}_{b}$ and $\Delta {h}_{4}$ are determined in the same way using Eqs. (S.3), (5), (6), (8), (9) and (16). Note that a final graph of ${R}_{b}$ vs $y$ is shown in Fig. S2 in the Supplementary Information file. This so called “root graph” is used to determine the roots at each instant. With ${R}_{t}$, ${R}_{b}$, $\Delta {h}_{3}$, $\Delta {h}_{4}$, $\Delta {h}_{1}$ and $\Delta {h}_{2}$ determined at this instant, “velocity Verlet” is applied to solve the equation of motion. The algorithm has predictor and corrector stages. For the predictor stage, the displacement of the particle $y(t)$ and its velocity $v(t)$ are predicted as:

17 $${y}^{p}\left(t+\delta t\right)= y\left(t\right)+\delta tv\left(t\right)+\frac{1}{2}\delta {t}^{2}a\left(t\right),$$

18 $${v}^{p}\left(t+\frac{1}{2}\delta t\right)= v\left(t\right)+\frac{1}{2}\delta ta\left(t\right),$$

where $\delta t$ is the time step of the simulation and a superscript $p$ denotes the predictor stage. For the corrector stage, the acceleration $a(t)$ or $\ddot{y}(\mathrm{t})$ of the particle is calculated using (1) in the form:

19 $$ma\left(t+\delta t\right)= F\left({y}^{p},{v}^{p}\right),$$

where $F({y}^{p},{v}^{p})$ is the force exerted on the particle. The velocity is then corrected $({v}^{c})$ based on the predicted velocity and acceleration.

20 $${v}^{c}\left(t+\delta t\right)= {v}^{p}\left(t+\frac{1}{2}\delta t\right)+\frac{1}{2}\delta ta\left(t+\delta t\right).$$

The corrected velocity and acceleration are used to predict the displacement of the particle $y$ in the next time step, and $y$ is used in Eqs. (S.2) and (S.3) in the first step of the solving procedure, which is iteratively repeated. Note that the calculation (19) requires roots ${R}_{t}$ and ${R}_{b}$. These are taken from the root graph (e.g. Fig. S2) at each instant.

## Results and discussion

### Effects of hydrogen bonding

**Figure 2:**
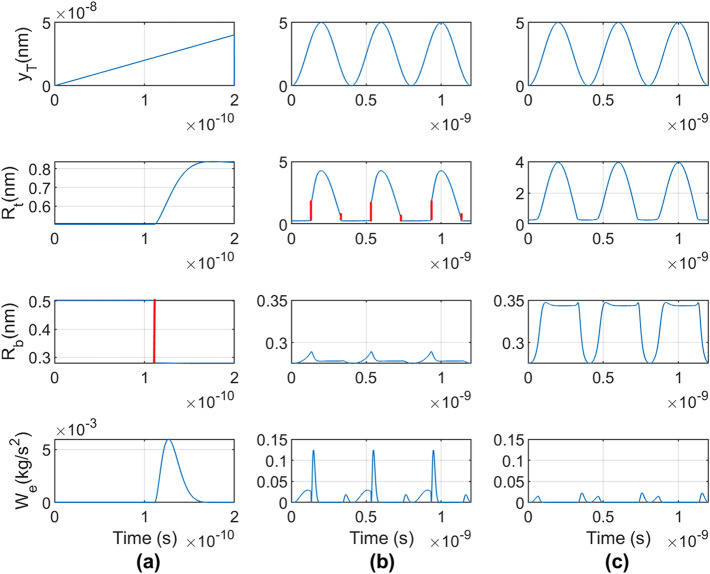
Displacement input ${y}_{T}$ and resulting response ${R}_{t}$ and ${R}_{b}$, and kinetic energy ${W}_{e}$ of the model. (**a**) Sinusoidal input displacement at 80 Hz (appears linear over this very short time); (**b**) sinusoidal input at 2.5 GHz; (**c**) sinusoidal input at 2.5 GHz with hydrogen bonding omitted from the model.

Simulations are conducted on the model with the bottom support (bottom matrix material) fixed. The response of the system is determined for various cases of prescribed motion of the top support (top matrix material). To create a similar dynamic condition as damping tests conducted by the authors^1^, Fig. 2a treats sinusoidal vibration of the top surface at a frequency of 80 Hz, which is selected from the average natural frequency of the CNC/PA610 samples in the aforementioned damping test measurements.

Plots in Fig. 2a show a sudden and rapid change in intermolecular distance ${R}_{b}$ (shown in red) of the bottom contacting surfaces, as well as an attendant increase in kinetic energy and intermolecular distance change ${R}_{t}$. The increase of the kinetic energy is due to increase in velocity of the mass brought upon by the imbalance of the forces exerted on its top and the bottom surfaces. The viscous damper in the model dissipates kinetic energy gained during the sudden intermolecular change in force, which is shown by the decrease of the maximum kinetic energy.

**Figure 3:**
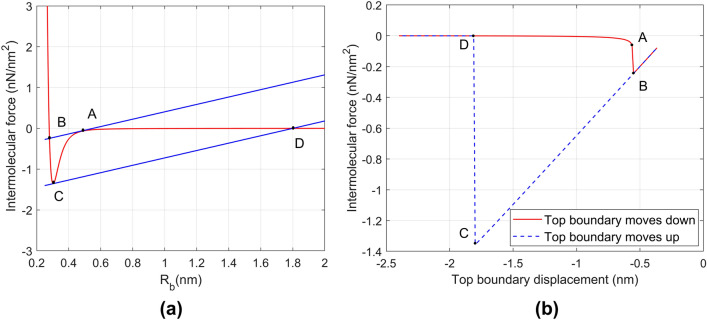
(**a**) Total bottom intermolecular force per unit area (red curve) vs intermolecular distance. The total elastic force per unit area in two instances are given by the blue curves. (**b**) Loading–unloading curve in massless and damper-less version of Fig. 1a showing hysteresis caused by the sudden intermolecular changes in force in (**a**) with same points A, B, C, D indicated.

Figure 3a presents the mechanism of the sudden intermolecular change in force in the case of Fig. 2a. When the top surface moves down, the intermolecular distances decrease. For a massless system^19^, it is shown that sudden intermolecular change in force can occur when the slope of the total elastic force equals the slope of the total intermolecular force. In Fig. 3a, this is possible at Points A and C and would result in sudden changes of force from A to B and C to D. For example, as ${y}_{T}$ increases, ${R}_{b}$ decreases from its initial value of 2 nm and moves towards point A where the intermolecular force “jumps” to point B. Similar behavior can occur from C to D if ${R}_{b}$ later increases.

In Fig. 3b, the same simulation as Fig. 3a is conducted for the case of no mass and no damper. It can be seen in the figure that the sudden intermolecular changes in force produce hysteresis in the system and so are irreversible. The hysteresis loop is therefore entirely due to the sudden intermolecular changes in force. Hysteresis loops due to this phenomena for the Maugis–Dugdale model are given in study^27^.

To demonstrate the effect of hydrogen bonding and multiple intermolecular changes of force on the interaction of these materials, results for the case of an input sinusoidal vibration of ${y}_{T}$ at 2.5 GHz for three cycles of the vibration are given in Fig. 2b, which includes hydrogen bonding, and Fig. 2c in which hydrogen bonding is omitted. This frequency is selected for convenience since it allows the model to run in a reasonable amount of computational time. As the input displacement ${y}_{T}$ cycles up and down, Fig. 2b shows sudden increases of intermolecular distance ${R}_{t}$ each cycle (shown in red), whereas these changes of forces do not occur in the case of Fig. 2c. This is due to hydrogen bonding in Fig. 2b increasing the intermolecular force and shifting it into a range where sudden intermolecular changes in force must occur for the elastic forces to balance the intermolecular forces. The sudden intermolecular changes in force in Fig. 2b produce sudden increases of the kinetic energy, and the peak kinetic energy of the particle in Fig. 2b is seen to be 455% greater than that in Fig. 2c.

**Figure 4:**
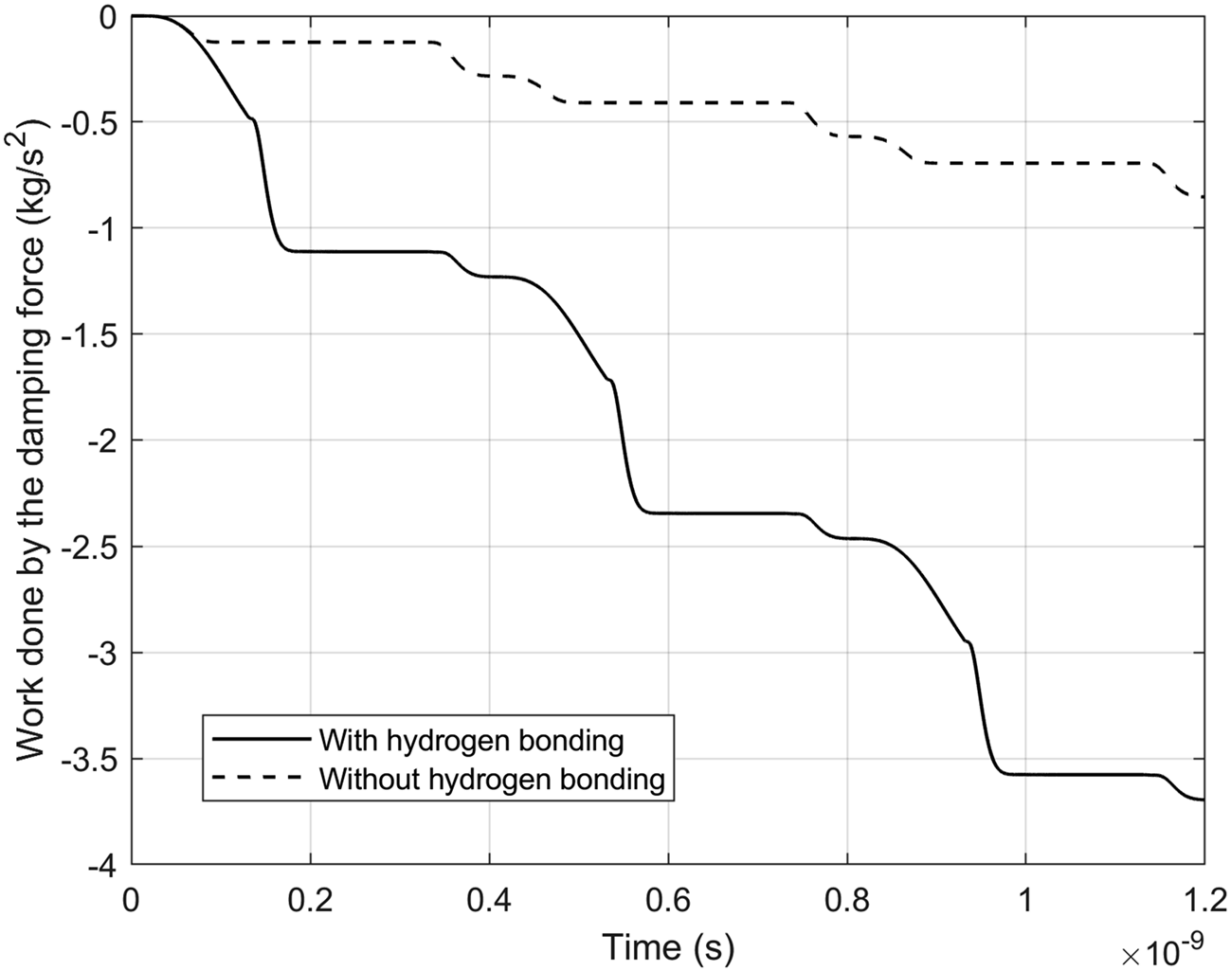
The work done by the damping force (per unit area) with and without hydrogen bonding participating in the simulations in Fig. 2b,c.

The work done by the damping force in the cases with and without hydrogen bonding is shown in Fig. 4 as the process progresses. The figure shows that the transfer of potential energy to kinetic energy in Fig. 2b results in an increase in energy dissipation by viscous damping, compared to Fig. 2c. Specifically, the work done by the damping force is 360% greater in the hydrogen bonding case compared to Fig. 2c where hydrogen bonding is omitted.

The results given here show the potential for hydrogen bonding between materials to increase damping. Hydrogen bonding has also been shown to increase damping in the context of interfacial friction^15,16^. Also, high damping was recently measured for bacteriological cellulose^40^ using DMA. In addition to hydrogen bonding, fiber alignment and ionic liquid were found to strongly affect damping^40^.

### Effects of parameters

**Figure 5:**
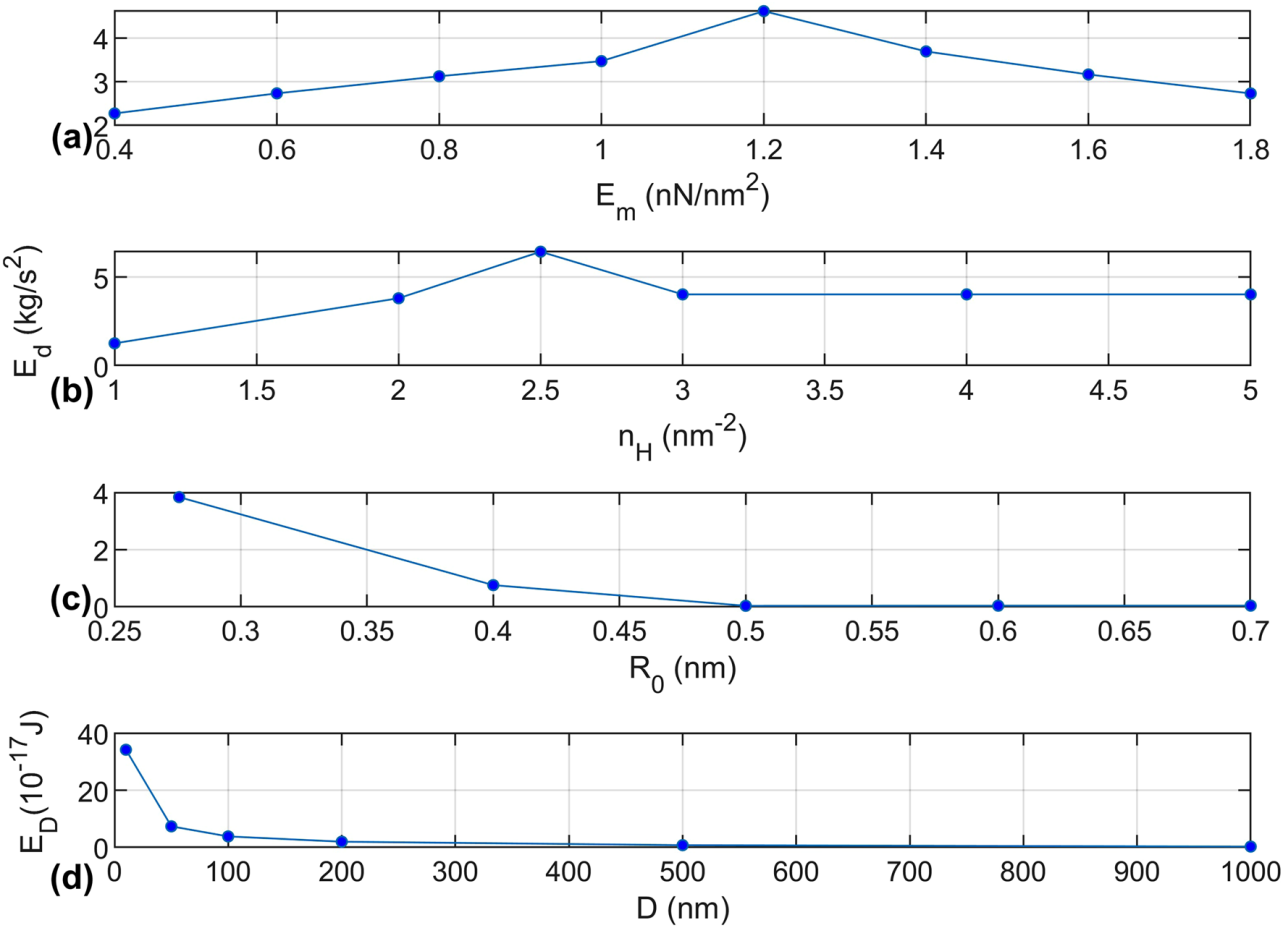
Energy dissipation after 3 cycles of the top material displacement (same input as in Fig. 2b) in terms of: (**a**) elastic modulus of matrix material for $D$ = 100 nm, ${n}_{H}$ = 2 nm^−2^, ${R}_{0}$ = 0.276 nm, ${E}_{p}$ = 145 nN/nm^2^; (**b**) hydrogen bonding density for $D$ = 100 nm, ${R}_{0}$ = 0.276 nm, ${E}_{m}$ = 1.4 nN/nm^2^, ${E}_{p}$ = 145 nN/nm^2^; (**c**) initial intermolecular distance for $D$ = 100 nm, ${n}_{H}$ = 2 nm^−2^, ${E}_{m}$ = 1.4 nN/nm^2^, ${E}_{p}$ = 145 nN/nm^2^; (**d**) size of the particles for ${n}_{H}$ = 2 nm^−2^, ${R}_{0}$ = 0.276 nm, ${E}_{m}$ = 1.4 nN/nm^2^, ${E}_{p}$ = 145 nN/nm^2^. Note that in (**a–c**), the energy dissipation is given per unit area.

Figure 5 shows the energy dissipated from the system as functions of the elastic modulus of the matrix material, ${E}_{m}$, the hydrogen bond density, ${n}_{H}$, the initial intermolecular distance, ${R}_{0}$, and the size of the particle, $D$. To study the effect of the relative stiffness of the contacting surfaces on the energy dissipation, the elastic modulus of the matrix material is varied from 0.4 to 1.8 nN/nm^2^ in Fig. 5a. Only ${E}_{m}$ is varied here for expediency. The range of ${E}_{m}$ selected represents ${E}_{p}$/${E}_{m}$ from 81 to 362, which covers a reasonable range of filler/matrix combinations. Energy loss in the system peaks at ${E}_{m}$ = 1.2 nN/nm^2^. This trend is primarily due to the intermolecular change in force that occurs in the vicinity of ${E}_{m}$ = 1.2 nN/nm^2^. Thus, the result shows that energy dissipation in particle filled composites may be maximized for a specific range of stiffnesses of the contacting materials, and this may be influenced by the occurrences of intermolecular changes of force.

**Figure 6:**
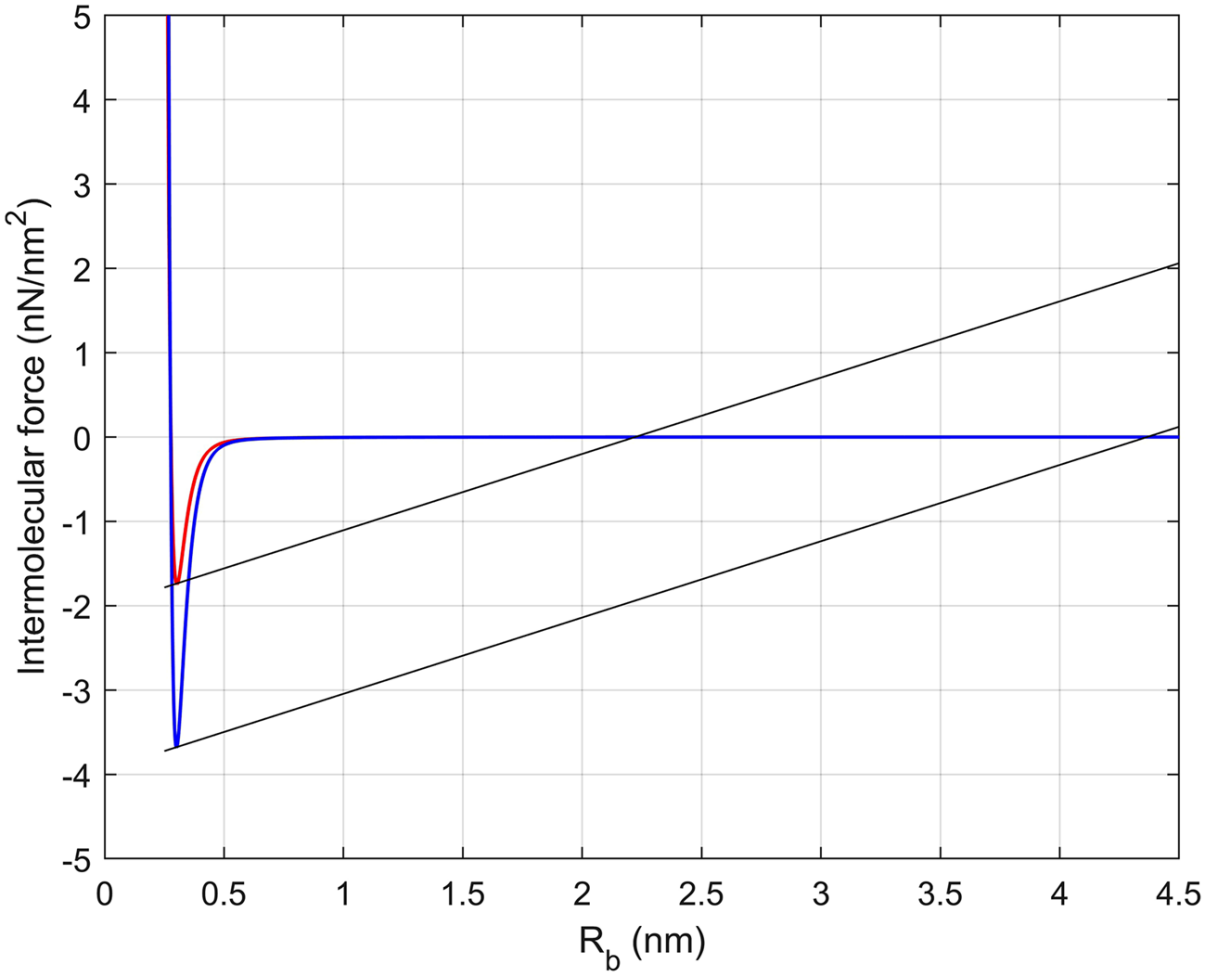
Intermolecular forces-distance graph. Two intermolecular forces (per unit area) at hydrogen bonding density: 2.5 nm^−2^ (red) and 5 nm^−2^ (blue).

Figure 5b shows that energy dissipation of the system peaks when hydrogen bond density is 2.5 nm^−2^, and then plateaus as density is increased further. When the hydrogen bond density is lower than 2.5 nm^−2^, there are two general reasons that the energy dissipation is constrained. First, the intermolecular change in force may not occur when the hydrogen bond density is too low because the condition described in relation to Fig. 3a may not be met. Second, the total kinetic energy of the particle gained during the change in force may be small due to the weak hydrogen bond strength. As for hydrogen bond density larger than 2.5 nm^−2^, Fig. 6 shows two intermolecular force curves at different hydrogen bond densities for the case where the top input amplitude ${y}_{T}$ is set to be the same (5 nm) in both cases. It is clear that a smaller hydrogen bond density can result in a smaller intermolecular change in force (from ${R}_{b}$ = 0.303 to 2.221 nm) than would a larger density (from ${R}_{b}$ = 0.301 to 4.366 nm). However, because of the strength of the intermolecular bonds in the larger density case, the maximum top and bottom intermolecular distance that occurs is 0.282 nm, so the intermolecular change in force at 0.301 nm does not occur. Therefore, energy dissipation is not necessarily maximum for higher bond densities, but rather occurs over an intermediate range for this composite.

Figure 5c shows that the energy loss is significantly lower for larger initial intermolecular distance ${R}_{0}$, the first point of which in the figure is ${R}_{H}$, the equilibrium distance given in Table 1. The decreasing trend of the energy loss is associated with the intermolecular change in force. In the case of the same input amplitude of the displacement ${y}_{T}$, the points where intermolecular changes in force can occur are not reached in the case of large initial ${R}_{0}$. Note that they could be reached if the range of ${y}_{T}$ were very large. This corresponds with damping test results conducted by the authors which show the damping ratios of this class of composites decrease after moisture in the composites is driven off^1^. Due to different rates of shrinkage of the filler and matrix materials, the process of drying will increase the free volume between the filler and matrix material^41^ so that the intermolecular distance will increase and possibly prevent adhesion.

In Fig. 5d, the energy dissipation for a fixed volume of particles is calculated as a function of the particle size $D$. In this case, the energy dissipation value is not per unit area. The total energy dissipation is calculated based on that of a single particle times the number of particles in a 1000 nm^3^ volume. The number of particles increases with the decrease of particle size $D$. It is noted that the total interfacial area increases with the decrease of particle size in the total fixed volume. When the size of the particles increase, it is seen that the energy dissipation significantly decreases. This trend corresponds to results from our damping measurements of CNC reinforced PA610 composites which show a strong increase in damping ratio of the composites as cellulose filler size decreases^1^, and is due to the increase of the contact surface area.

The model introduced here offers several advancements to previous one-dimensional adhesion models. In studies^19,42^, both models are based on a massless contacting system with van der Waals force and a simple elastic spring force model. The work in study^21^ advances these models to solid–liquid interface cases with adhesion hysteresis and friction, indicating the possibility for sudden intermolecular changes in force and their relation to irreversible processes and dissipation in a conservative system. The present intermolecular contact model is intended to represent a nanoscale filler particle in a matrix carrier, including the mass of the particle, hydrogen bonding between the contacting surfaces, and a contact force based on surface roughness^27,31^. A key finding is that hydrogen bonding at the interface can promote sudden intermolecular force changes that may increase damping. Based on the study of this model, the substantial damping improvement from CNC particles is believed to be due to high surface area and occurrences of intermolecular changes in force in the presence of hydrogen bonding.

The intermolecular contact model developed in this study is one-dimensional. This necessarily neglects the effects of more complex deformations on the damping. Additionally, the contacting materials are assumed to be well-bonded, so interfacial slip^15^ is not included. Multiple bonds are included through the effect of bond density per unit area which is not as general as a full molecular dynamics simulation. For composite systems with strong bonds, such as covalent bonds, between filler and matrix, the intermolecular forces either restrict movement at the interfaces or are too strong to occur in a range where sudden intermolecular changes in force must occur for the elastic forces to balance the intermolecular forces. To be applicable to covalently bonded systems, the present model would require modification of the forcing functions. Despite these limitations, the model captures the interrelated effects of hydrogen bonding and sudden intermolecular changes in force on energy dissipation in a straightforward context, and forms the basis for more general multi-dimensional studies.

## Conclusion

In this study, an interfacial dynamics model for the filler and matrix contacting surfaces of a particle reinforced composite is developed. In the case of cellulose reinforced PA610 composites, the elastic force from asperities of the contacting surfaces and intermolecular hydrogen bonding encourage sudden intermolecular changes in force. The simulation results show strong relationship between the hydrogen bonding at the interface and increase of energy dissipation that qualitatively corresponds to previously measured damping test results. The energy loss is in negative proportion to the particle size and initial intermolecular distance. Additionally, there are optimal values of the hydrogen bonding density and elastic modulus ratio of the materials that increase the energy loss. The model can be used to evaluate designs of composites seeking to maximize energy loss.

## Supplementary Information

Supplementary Information.

## Data Availability

The data analyzed in this article are included in the main text and the Supplementary Information file.
