# Supplementary material for: Effects of interfacial dynamics on the damping of biocomposites
Source: Sci Rep. 2022 Nov 21;12:20042. doi: 10.1038/s41598-022-23355-x (PMC9681862; doi:10.1038/s41598-022-23355-x)
Supplement: Supplementary file 1 — Supplementary Information. [file 41598_2022_23355_MOESM1_ESM.docx]

**Effects of Interfacial Dynamics on the Damping of Biocomposites**

**Supplementary Information**

Yufeng Tian^a^, Wonsuk Kim^a^, Alper Kiziltas^b^, Deborah Mielewski^b^, Alan Argento^a,^*

^a^ University of Michigan-Dearborn, Department of Mechanical Engineering, 4901 Evergreen Road, Dearborn, MI, 48128, United States

^b^ Ford Motor Company, Sustainability and Emerging Materials, Dearborn, MI, 48128, United States

* Corresponding author, [aargento@umich.edu](mailto:aargento@umich.edu)

The coordinates and deformations of the springs in Fig. 1(a) are related to the intermolecular distance through simple geometry of the model. For the top part of the model above the mass:

| $y-y_{T}\left( t \right)= \left( \Delta h_{1}-\Delta h_{01} \right)+(\Delta h_{3}-\Delta h_{03})+(R_{t}-R_{0})$ | (S.1) |
| --- | --- |

where $R_{0}$,$R_{t}$ are the initial and current intermolecular distances, respectively, $\Delta h_{1}$and $\Delta h_{3}$ are the current deformations of the elastic elements of the particle (above the mass) and top matrix material, respectively. $\Delta h_{01}$ and $\Delta h_{03}$ are the initial deformations of the elastic elements of the particle (above the mass) and top matrix material, respectively. Note that $R_{0}, \Delta h_{01}, \Delta h_{03}$ are given constants and $y_{T}\left( t \right)$ is a prescribed function. Equation (S.1) can be simplified as:

| $R_{t} = R_{0}+\Delta h_{01}+\Delta h_{03}-\Delta h_{1}-\Delta h_{3}+y-y_{T}(t)$ | (S.2) |
| --- | --- |

Equation (S.2) relates the current intermolecular distance to the current deformations of the elastic elements. Similarly, for the bottom part of the model, the relationship for the intermolecular distance $R_{b}$ is:

| $R_{b} = R_{0}+\Delta h_{02}+\Delta h_{04}-\Delta h_{2}-\Delta h_{4}-y$ | (S.3) |
| --- | --- |

Where, $\Delta h_{02}$ and $\Delta h_{04}$ are the initial deformations of the elastic elements of the particle (below the mass) and bottom matrix material, respectively. $\Delta h_{2}$ and $\Delta h_{4}$ are the current deformations of the elastic elements of the particle (below the mass) and the bottom matrix material, respectively.

In Fig. S.1, the initial positions of the donor of the hydrogen atom, the hydrogen atom, and the acceptor of hydrogen atom are respectively shown as A, B, C, and are connected by solid lines. $\theta$*,*$\alpha$*,* $\beta$ are the angles between these atoms. $L_{ab}$ and $L_{bc}$ are the initial bond lengths between the atoms at A, B, C, as shown. $R_{i}$ is the initial hydrogen bonding length. When the hydrogen bond increases to current length $R$, the atom at C moves to current position D (assuming A is fixed). For the case of adhesion, the lateral movement of atom C as it moves to point D will not be considered.


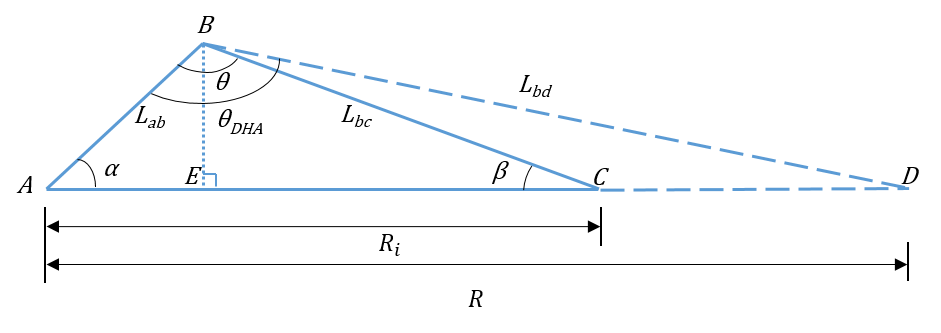


**Figure S.1**. Schematic for the hydrogen bonding angle, $\theta_{DHA}$.

The relationship between the angles at the initial position is:

| $\frac{R_{i}}{sin\theta}= \frac{L_{ab}}{sin\beta}= \frac{L_{bc}}{sin\alpha}$ | (S.4) |
| --- | --- |

The relationship between the angle $\theta_{DHA}$and side lengths at the current position is:

| $cos\theta_{DHA}= \frac{L_{ab}^{2}+L_{bd}^{2}-R^{2}}{2L_{ab}L_{bd}}$ | (S.5) |
| --- | --- |

$L_{bd}$ is determined by:

| $L_{bd}= \sqrt{{(L}_{ab}{sin\alpha)}^{2}+{(R-L}_{ab}{cos\alpha)}^{2}}$ | (S.6) |
| --- | --- |

Using equation (S.4)-(S.6), $cos\theta_{DHA}$ can be obtained as:

| $cos\theta_{DHA}= \frac{L_{ab}^{2}+{(sin\alpha\cdot L_{ab})}^{2}+{(R-cos\alpha\cdot L_{ab})}^{2}-R^{2}}{2L_{ab}\sqrt{{(sin\alpha\cdot L_{ab})}^{2}+{(R-cos\alpha\cdot L_{ab})}^{2}}}$ | (S.7) |
| --- | --- |

Figure S.2 describes the locus of roots $R_{b}$ of equation (5). Since the materials of the top contacting surfaces are the same as the bottom ones, the shape of the root graphs for $R_{b}$ and $R_{t}$ are identical. Each point on the curve represents a root of equation (5) at a time step. To better explain how the roots are selected, the initial intermolecular distance is set to be 0.276 nm. Note that the positive direction of $y$ is downward. As $y$ deceases from 0, the intermolecular distance $R_{b}$ follows the root curve until $y$ reaches -1.79 nm, where $R_{b}$=0.3 nm. When $y$ moves further to the left beyond this point -1.79 nm, the continuing root $R_{b}$ disappears and suddenly changes to 1.8 nm. It then follows the root curve from this point. This process is indicated by the direction of the blue arrows. A similar process occurs when the surfaces approach each other, which is shown in the direction of the black arrows. The root results are used in the model to determine the intermolecular force.


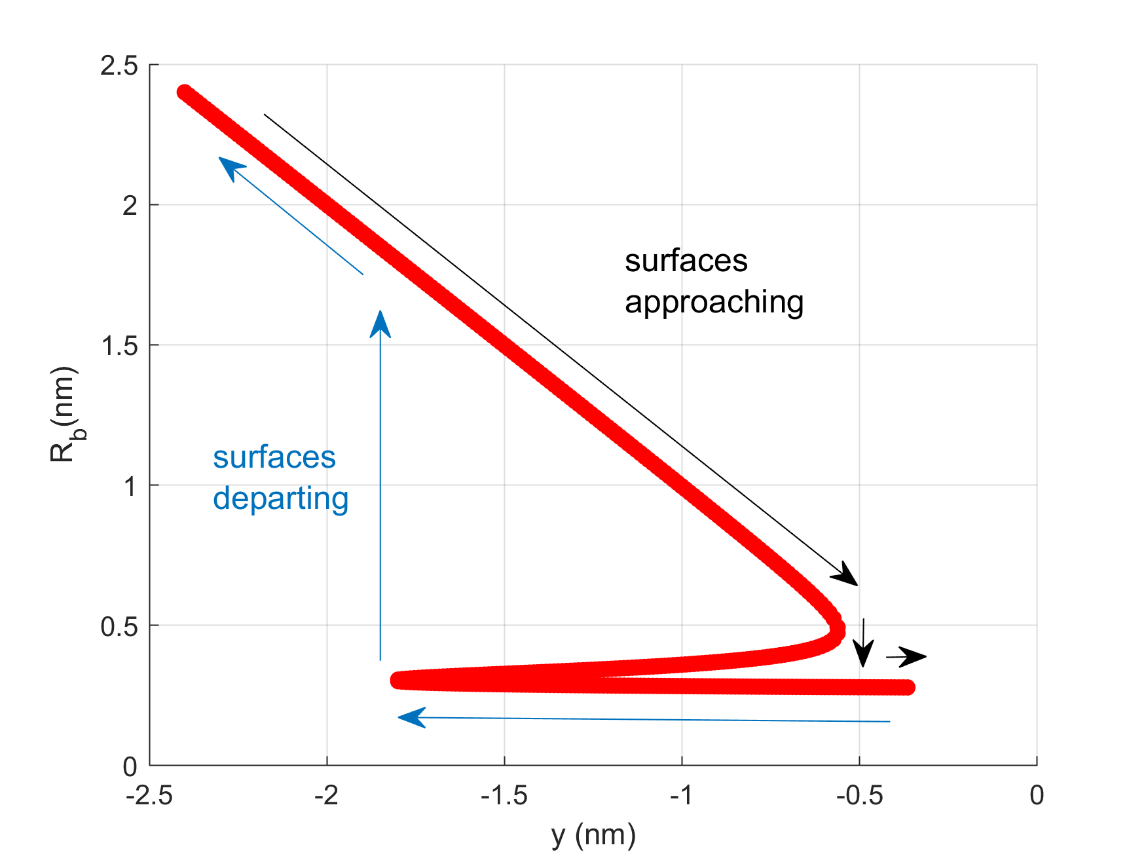


**Figure S.2**. Intermolecular distance, $R_{b}$, of equation (5) vs displacement $y$ of the particle.
